# Weiterbildungsordnung – in welche Richtung geht es?

**DOI:** 10.1007/s00117-026-01655-x

**Published:** 2026-08-13

**Authors:** Isabel Molwitz, Eva See, Konstantin Nikolaou, Fabian Bamberg

**Affiliations:** 1 Department of Diagnostic and Interventional Radiology and Nuclear Medicine, University Medical Center Hamburg-Eppendorf, Martinistr. 52, 20246 Hamburg, Deutschland; 2 Department of Radiology, Massachusetts General Hospital, Harvard Medical School, Boston, USA; 3 radiomedicum – Gemeinschaftspraxis für Radiologie und Nuklearmedizin, Frankfurt, Deutschland; 4 Department of Diagnostic and Interventional Radiology, University of Tuebingen, Tübingen, Deutschland; 5 Department of Radiology, Medical Center and Faculty of Medicine, University of Freiburg, Freiburg, Deutschland

Auf dem diesjährigen Deutschen Ärztetag (DÄT) standen erste Aspekte der bereits durch den 128. DÄT 2024 beschlossenen Überarbeitung der Musterweiterbildungsordnung (MWBO) zur Abstimmung. Dieser Artikel gibt einen Überblick über die in den vergangenen zwei Jahren thematisierten Novellierungen für den Fachbereich der Radiologie inklusive der Schwerpunkte Kinder- und Neuroradiologie, Einblicke in den 130. DÄT im Mai 2026 sowie Informationen zu perspektivisch anstehenden weiteren Änderungen.

## Seit 2024 bereits erfolgte Novellierungen der MWBO

Auf dem 128. DÄT wurden eine Aktualisierung und Verschlankung der Inhalte der Facharztweiterbildungen, die Prüfung einer Verkürzung der Weiterbildungszeit, eine Überarbeitung des Paragraphenteils und eine Überprüfung der Zusatzweiterbildungen beschlossen [1].

Im ersten Schritt wurden seitens der Bundesärztekammer (BÄK) die Zusatzweiterbildungen überarbeitet. Konkret ging es um die Zuordnung zu verschiedenen Kategorien (C1: hauptberuflich und die gesamte Arbeitszeit einnehmend; C2: berufsbegleitend mit Weiterbildungsprüfung; C3: Kurs ohne Weiterbildungszeiten). Weiterhin wurde geprüft, ob einzelne Zusatzweiterbildungen eher als Schwerpunkte einzuordnen sind, was im Gegensatz zu einer Zusatzweiterbildung zunächst das Vorhandensein spezifischer Facharztgebiete erfordert. Ebenso wurde evaluiert, ob einige Zusatzweiterbildungen gar keine Aufführung in der MWBO erfordern und stattdessen Zertifizierungen durch Fachgesellschaften ausreichend wären, und es wurden mögliche neue Zusatzweiterbildungen geprüft.

Für die Radiologie relevant war primär die Überprüfung der Zusatzweiterbildung „Kardiale Magnetresonanztomographie“. Im Gegensatz zu einem Schwerpunkt ist diese als Zusatzweiterbildung auch anderen Fachgebieten zugänglich. Die insbesondere seitens der Kardiologie gewünschte Änderung auf die Kategorie C3 (nur kursbasiert ohne Weiterbildungszeiten) konnte in Anhörungen in der BÄK anhand des damit einhergehenden deutlichen Qualitätsverlustes abgelehnt werden. Die Zusatzweiterbildung „Kardiale Magnetresonanztomographie“ verbleibt somit der Kategorie C2 zugeordnet und erfordert weiterhin eine durch einen Weiterbildungsbefugten für Herz-MRT bestätigte Weiterbildungszeit sowie eine Prüfung. Auch die Zusatzweiterbildungen „Nuklearmedizinische Diagnostik für Radiologen“ und „Röntgendiagnostik für Nuklearmediziner“ konnten unverändert erhalten werden.

## Weitere Forderungen der Radiologie und ihrer Schwerpunkte

Im Anschluss beschäftigte sich die BÄK mit der Novellierung der Abschnitte A (Paragraphenteil) und B (Facharztgebiete und Schwerpunkte) der MWBO. Hierzu wurde seitens der Radiologie eine Vorstandskommission gebildet, in der Vertreterinnen und Vertreter der Deutschen Röntgengesellschaft (DRG), des Berufsverbands (BDR), der Kinder- und Jugendradiologie (GPR), der Neuroradiologie (DGNR) und der Deutschen Gesellschaft für Interventionelle Radiologie und minimal-invasive Therapie (DeGIR) gemeinsam für den Fachbereich relevante Themen bearbeiteten. Durchgehend eingebunden waren auch jeweils Vertreterinnen und Vertreter der Nachwuchsorganisationen. Überdies führte das Forum Junge Radiologie (FJR) im März 2025 eine Umfrage durch, um sicherzustellen, dass die Wünsche der Weiterzubildenden adäquat abgebildet würden [2].

Die für den Paragraphenteil formulierten Forderungen dienten insbesondere dazu, die Inklusion von Menschen mit Beeinträchtigungen zu fördern. So sollte eine Beeinträchtigung nicht zu einer Benachteiligung hinsichtlich Weiterbildungszeit und -qualität führen. Weiterhin forderten wir in Hinblick auf den steigenden Anteil von Kolleginnen und Kollegen in Teilzeit, eine Abkehr von der bisher geltenden Untergrenze von 50 % Teilzeit und stattdessen die Anrechnung jeglicher Tätigkeit in Teilzeit auf die Weiterbildung, auch bspw. einer 20 % Teilzeittätigkeit.

Für den Abschnitt B, der sich auf Facharztgebiete und ihre Schwerpunkte bezieht, ergab sich unter Berücksichtigung der FJR-Umfrageergebnisse [2] entgegen dem Vorschlag der BÄK nach 4‑jähriger Weiterbildungsdauer der Wunsch, eine 5‑jährige Weiterbildungsdauer sowie 24-monatige Schwerpunktweiterbildungszeit beizubehalten. Weiterhin wurden Fallzahlen von den Weiterzubildenden nach wie vor gewünscht, wenn auch mit Fokus auf den Kompetenzerwerb. Auch die zunehmende Integration und Anrechenbarkeit von Fallsammlungen und Simulatorkursen für die Weiterbildung wurden als sinnvoll erachtet, ebenso wie ein zusätzlicher Schwerpunkt für interventionelle Radiologie. Digitale Kompetenzen insbesondere auch zu künstlicher Intelligenz sollten neu im radiologischen Fachgebiet aufgeführt und der Strahlenschutz gestärkt werden. Ein weiterer wichtiger Punkt betraf die Anerkennung von in den radiologischen Schwerpunkten oder der patientennahen Forschung geleisteten Zeiten auf die Facharztweiterbildung Radiologie, äquivalent zur bisher gegebenen Anrechnung von bis zu 12 Monaten Weiterbildungszeit in anderen Fachgebieten. Seitens der, aufgrund geringer Absolventenzahlen des Schwerpunkts notwendigen, Stärkung der Kinder- und Jugendradiologie wurde die Untersuchung von Patientinnen und Patienten jeglichen Alters in die geforderten Fallzahlen innerhalb des Fachgebiets Radiologie integriert.

## Entscheidungsprozess im Vorfeld des 130. DÄT

Nach Anhörung der Fachvertreterinnen und -vertreter in der BÄK im Herbst 2025 wurde der Integration digitaler Kompetenzen in das Fachgebiet der Radiologie sowie der Stärkung von Fallsammlungen und Simulatorkursen zugestimmt. Auch die Vorschläge zum Strahlenschutz wurden übernommen, ebenso wie die Forderung, Untersuchungen von Patientinnen und Patienten jeglichen Alters für die Fallzahlen des Fachgebiets der Radiologie zu erbringen. Die Anrechenbarkeit von Zeiten in den Schwerpunkten oder der Forschung auf die Weiterbildungszeit des Facharztgebiets Radiologie wurde hingegen grundsätzlich abgelehnt. Hinsichtlich der Forschung wurde auf durch die Kammern genehmigte Clinician-Scientists-Programme verwiesen. Ein möglicher zusätzlicher Schwerpunkt „Interventionelle Radiologie“ wurde durch die BÄK für einen späteren Zeitpunkt vermerkt.

Ende des Jahres entschied die Ständige Konferenz für Ärztliche Weiterbildung (StäKo) jedoch, entgegen den Forderungen bspw. auch der Vertreterinnen und Vertreter der Radiologie, eine Kürzung der Weiterbildungszeit von 5 auf 4 Jahre für zahlreiche Facharztgebiete. In konzertierter Abstimmung mit anderen jungen Fachgesellschaften unter aktiver radiologischer Beteiligung konnte das Bündnis Junge Ärztinnen und Ärzte dem jedoch in einem Positionspapier widersprechen [3].

## Entwicklungen rund um die Weiterbildung auf dem 130. DÄT

**Abb. 1:**
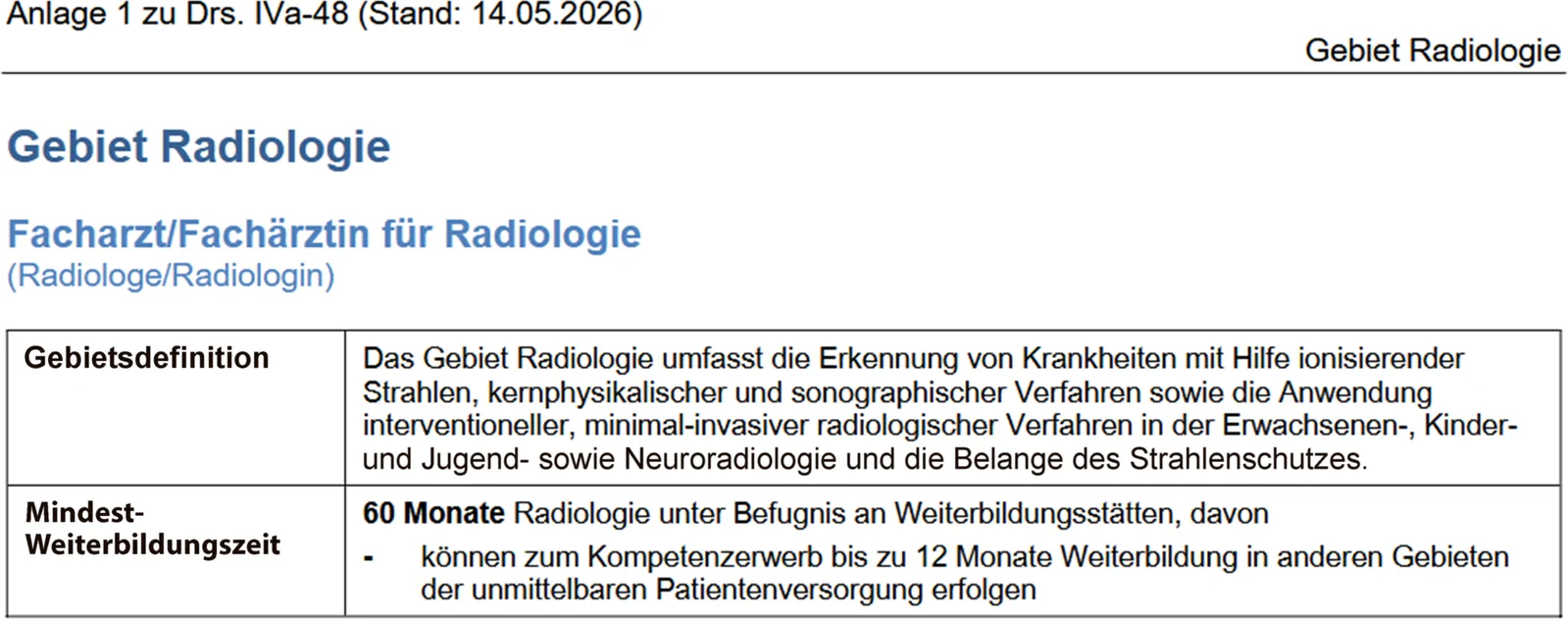
Beschlossene Änderung des Kopfteils der MWBO für das Gebiet Radiologie [5]

Der 130. DÄT im Mai 2026 beschloss mit dem Antrag IVa – 48 für die Facharztweiterbildung Radiologie eine Mindestweiterbildungszeit von 60 Monaten sowie entsprechend des Wunsches der Kinderradiologie eine Umbenennung in Kinder- und Jugendradiologie, wie in Abb. 1 dargestellt [4].

Als Delegierte für die Landesärztekammer Hessen brachte Dr. Eva See zudem gemeinsam mit Kolleginnen und Kollegen aus anderen Fachgebieten zwei Anträge (IVc-41; Ic-71) zur Stärkung der Weiterbildung in der Radiologie ein. So forderte Antrag IVc – 41 eine bundesweit einheitliche Anrechenbarkeit von Weiterbildungszeiten in den Schwerpunkten des Gebiets der Radiologie auf die Facharztweiterbildung für Radiologie [6]. Konkret wird im Antrag an die Landesärztekammern appelliert, Weiterbildungsbefugnisse im Gebiet der Radiologie so auszugestalten, dass bei Vorliegen der strukturellen, personellen und inhaltlichen Voraussetzungen auch in Schwerpunkten erworbene Weiterbildungszeiten anteilig auf die Facharztweiterbildung Radiologie angerechnet werden können. Der Antrag soll Weiterbildungskapazitäten besser nutzen, die Qualität der Weiterbildung stärken, Assistenzärztinnen und -ärzten frühzeitige Einblicke in die Schwerpunkte erleichtern und somit dem Nachwuchsmangel insbesondere auch in der Kinderradiologie entgegenwirken. Aus organisatorischen Gründen erfolgte die Überweisung dieses und weiterer Anträge an den Vorstand der BÄK.

In dem mit großer Mehrheit angenommenen Beschlussantrag Ic-71 forderte der 130. DÄT 2026 zudem die BÄK und das Bundesministerium für Gesundheit dazu auf, gemeinsam mit der Selbstverwaltung, den Kassenärztlichen Vereinigungen und den Ländern die Rahmenbedingungen für die ärztliche Niederlassung zu überprüfen und weiterzuentwickeln [7]. Ziel ist, dass auch für zukünftige Generationen von Ärztinnen und Ärzten eine realistische Perspektive besteht, fachärztliche Einzel- oder Gemeinschaftspraxen zu gründen bzw. zu übernehmen, wirtschaftlich tragfähig zu führen und zur wohnortnahen ambulanten Versorgung beizutragen. Konkret gefordert wurde u. a. die strukturelle Stärkung inhabergeführter, freiberuflicher Praxen – auch in technisch, interventionell und operativ geprägten Fachgebieten wie der Radiologie. Weiterhin enthielt der Antrag die Forderung nach gezielter Nachwuchsförderung einschließlich der Stärkung der Weiterbildung in der Niederlassung sowie geeigneter Finanzierungsinstrumente (z. B. Übernahmefinanzierung, Bürgschaften, Sicherstellungszuschläge) für junge Ärztinnen und Ärzte, die den Schritt in die Selbstständigkeit wagen wollen [7].

Auch der u. a. von Dr. Konstanze Schütze als Landesvorsitzende des BDR in Berlin eingebrachte Antrag IVc-49 forderte die Stärkung der ambulanten Weiterbildung in den Fachgebieten der unmittelbaren Patientenversorgung mit expliziter Nennung der primär in der Niederlassung vertretenen muskuloskeletalen Radiologie [8].

Auswirkungen auf alle Fachgebiete wird überdies der Beschlussantrag IVa-03 des Vorstands zur Neustrukturierung der Allgemeinen Inhalte der MWBO haben [9]. Dieser sieht die Neustrukturierung des bisher als Kompetenzen und Richtzahlen formulierten Abschnitts B (Facharztgebiete und Schwerpunkte) auf Basis der CanMEDS-Rollen (Kenntnisse, Haltungen, Rollen) vor [9].

## Perspektivisch anstehende weitere Novellierungen

Im Nachgang der bisherigen Novellierungen der MWBO ist seitens der BÄK die Beschäftigung mit zwischenzeitlich eingebrachten Vorschlägen zu neuen Schwerpunkten, so auch zur interventionellen Radiologie, und im Anschluss ggf. eine Überarbeitung des bestehenden Formats der Facharztprüfung geplant.

Ein entsprechend bereits eingereichter Vorschlag für einen Schwerpunkt „Interventionelle Radiologie“ folgt strukturell den bestehenden Schwerpunkten Kinder- und Neuroradiologie. Der Inhalt orientiert sich an der seit den 2010er-Jahren etablierten Qualitätssicherungs- und Zertifizierungssystematik der DeGIR mit ihren Modulen A bis E (bzw. F) und berücksichtigt zugleich die aktuelle Versorgungssituation sowie die notwendige Kohärenz zum Fachgebiet der Radiologie insgesamt. Begleitet wurde der Vorschlag von einer umfassenden Analyse zur Bedeutung der interventionellen Radiologie für die Patientenversorgung, ihrer bundesweiten Versorgungsabdeckung sowie zukünftiger Entwicklungsperspektiven.

Um die Facharztprüfung entsprechend der tatsächlichen Bedürfnisse der Weiterzubildenden zu überarbeiten, führt das FJR aktuell eine Umfrage zur Evaluation der bestehenden technischen, strukturellen und inhaltlichen Ausgestaltung der Facharztprüfung sowie möglicher gewünschter Veränderungen durch.

## Fazit

Die MWBO wird auch zukünftig kontinuierlich überarbeitet werden. Eine vereinte Interessenvertretung unseres Fachgebiets und seiner Schwerpunkte sowie, wenn möglich, auch gemeinsam mit Kolleginnen und Kollegen anderer Fachgebiete, ist essenziell, um aktuelle fachliche Entwicklungen effektiv einzubringen. Bedeutsam ist dabei vor allem auch das Engagement von Radiologinnen und Radiologen in den Ärztekammern sowie die Anwesenheit auf dem DÄT. So konnten zahlreiche Forderungen für das Fachgebiet der Radiologie und seiner Schwerpunkte in der aktuellen Überarbeitungsphase der Zusatz- und Gebietsweiterbildungen umgesetzt werden.

## Data Availability

Alle dieser Arbeit zugrunde liegenden Daten sind in diesem Artikel enthalten.
